# ω-3 Polyunsaturated fatty acids prevent pressure overload-induced ventricular dilation and decrease in mitochondrial enzymes despite no change in adiponectin

**DOI:** 10.1186/1476-511X-9-95

**Published:** 2010-09-06

**Authors:** Karen M O'Shea, David J Chess, Ramzi J Khairallah, Peter A Hecker, Biao Lei, Kenneth Walsh, Christine Des Rosiers, William C Stanley

**Affiliations:** 1 Division of Cardiology and Department of Medicine, University of Maryland, Baltimore, MD, USA; 2 Whitaker Cardiovascular Institute, Boston University School of Medicine, Boston, MA, USA; 3 Department of Nutrition and Montreal Heart Institute, Université de Montréal, Montreal, Canada

## Abstract

**Background:**

Pathological left ventricular (LV) hypertrophy frequently progresses to dilated heart failure with suppressed mitochondrial oxidative capacity. Dietary marine ω-3 polyunsaturated fatty acids (ω-3 PUFA) up-regulate adiponectin and prevent LV dilation in rats subjected to pressure overload. This study 1) assessed the effects of ω-3 PUFA on LV dilation and down-regulation of mitochondrial enzymes in response to pressure overload; and 2) evaluated the role of adiponectin in mediating the effects of ω-3 PUFA in heart.

**Methods:**

Wild type (WT) and adiponectin-/- mice underwent transverse aortic constriction (TAC) and were fed standard chow ± ω-3 PUFA for 6 weeks. At 6 weeks, echocardiography was performed to assess LV function, mice were terminated, and mitochondrial enzyme activities were evaluated.

**Results:**

TAC induced similar pathological LV hypertrophy compared to sham mice in both strains on both diets. In WT mice TAC increased LV systolic and diastolic volumes and reduced mitochondrial enzyme activities, which were attenuated by ω-3 PUFA without increasing adiponectin. In contrast, adiponectin-/- mice displayed no increase in LV end diastolic and systolic volumes or decrease in mitochondrial enzymes with TAC, and did not respond to ω-3 PUFA.

**Conclusion:**

These findings suggest ω-3 PUFA attenuates cardiac pathology in response to pressure overload independent of an elevation in adiponectin.

## Introduction

Consumption of diets enriched in ω-3 polyunsaturated fatty acids (ω-3 PUFA) from fish (eicosapentaenoic acid (EPA) and docosahexaenoic acid (DHA)) is strongly associated with a decreased risk of coronary heart disease and sudden cardiac death [1-3]. Recent evidence suggests that ω-3 PUFA from fish reduces the incidence of heart failure [4], and that dietary supplementation with a low dose of ω-3 PUFA decreases mortality and hospitalization in heart failure patients [5]. We previously reported that pretreatment with an ω-3 PUFA-enriched diet protects against ventricular remodeling and dysfunction in a model of moderate left ventricular (LV) hypertrophy and dysfunction induced by abdominal aortic banding in rats [6-9]. More severe LV hypertrophy caused by transverse aortic constriction (TAC) results in chamber dilation and a decrease in mitochondrial oxidative enzyme activities [10,11], which is associated with a decrease in the activity of the fatty acid-activated nuclear receptor peroxisome proliferator-activated receptor α (PPARα) [12]. The effects of ω-3 PUFA on the activation of PPARα or cardiac mitochondrial oxidative capacity have not been reported under conditions of severe hypertrophic heart failure. Thus it remains to be shown if part of the beneficial effect of ω-3 PUFA under conditions of chronic stress is mediated via maintenance of myocardial oxidative capacity.

The mechanisms responsible for the protective effects of ω-3 PUFA are not well-understood. We found that dietary supplementation with ω-3 PUFA elevated adiponectin concentration in the plasma in a dose-dependent manner in rats [7], indicating a potential mechanistic role for adiponectin in mediating the effects of ω-3 PUFA. Adiponectin is an insulin-sensitizing, anti-inflammatory hormone that is synthesized in adipocytes and can exert cardioprotective effects in many species, including mice [13]. An increase in circulating adiponectin with ω-3 PUFA feeding has been observed in mice [14,15]. In contrast, elevated circulating adiponectin is a strong positive predictor of mortality and poor outcome in heart failure patients [16-19]. Previous reports with short-term pressure overload (1-3 weeks) induced by transverse aortic constriction (TAC) showed that adiponectin-deficient (adiponectin-/-) mice displayed concentric LV hypertrophy [20], increased mortality [20,21] and lower ejection fraction compared to wild type (WT) mice [21]. On the other hand, we recently reported that adiponectin-/- mice subjected to 6 weeks of TAC displayed concentric hypertrophy, preservation of ejection fraction and chamber size, and no decrease in mitochondrial enzyme activities compared to WT mice [22]. At present, it is unclear if the beneficial effects of ω-3 PUFA in preventing the development and progression of heart failure are dependent upon an increase in circulating adiponectin.

The goals of the present study were to 1) assess the effects of ω-3 PUFA on LV chamber expansion and down-regulation of mitochondrial enzyme activities in mice subjected to TAC; and 2) evaluate the role of adiponectin in mediating the effects of ω-3 PUFA. Wild type and adiponectin-/- mice underwent sham surgery or TAC to elicit LV hypertrophy and heart failure, and were fed either a standard chow or chow supplemented with ω-3 PUFA for 6 weeks. The data from the standard chow-fed adiponectin-/- and WT animals from this study were previously published [22], and are included here to serve as controls. The development of heart failure was evaluated by changes in LV chamber size and up-regulation of the mRNA of fetal genes. To determine if ω-3 PUFA supplementation prevents down-regulation of oxidative capacity the activity of mitochondrial marker enzymes was measured. The role of adiponectin in mediating the effects of ω-3 PUFA was evaluated by measurement of serum adiponectin levels, and by performing a parallel series of experiments in adiponectin-/- mice.

## Methods

### Experimental Design

Investigators were blinded to treatment when measurements were performed. The animal protocol was conducted according to the Guideline for the Care and Use of Laboratory Animals (NIH publication No. 85-23) and was approved by the University of Maryland School of Medicine Institutional Animal Care and Use Committee. The animals were maintained on a 12-hour light-dark cycle and all procedures were performed between 3 and 6 hours from the start of the light phase. Adiponectin-/- mice were generated as previously described [23]. It is important to note that the data from the WT and adiponectin-/- mice without ω-3 PUFA supplementation were previously reported in a separate publication that presented the response between the two strains to pressure overload [22]. These studies showed that the adiponectin-/- mice had similar LV hypertrophy and increase in mRNA markers of heart failure as WT mice; however, adiponectin deletion prevented the increase in LV chamber size and the decrease in the activity of mitochondrial oxidative enzymes. In the present investigation we show these data again to allow comparison with the ω-3 PUFA treated groups. All experimental groups were contemporary.

Male C57BL/6J (WT) mice and adiponectin-/- mice (20-25 g) were subjected to TAC with a 27G needle as previously described [24] or sham surgery (n = 12-15/group). Two to three days after surgery, the mice were fed a standard chow (STD) or modified standard chow containing fish oil composed primarily of DHA and EPA. The mice were maintained on the diet for 6 weeks. Tail blood pressures were measured at 3-4 weeks using the tail cuff system. At 6 weeks, LV function was analyzed by echocardiography and mice were anesthetized with isoflurane, blood was drawn by cardiac puncture into the right ventricle, and the heart was harvested for biochemical analysis.

### Diets

Chows were custom-manufactured (Research Diets Inc. New Brunswick, NJ). The STD and ω-3 PUFA diets both derived 70% of total energy from carbohydrate (40% of total energy from cornstarch, 5% from maltodextrin and 25% from sucrose), 20% protein (casein supplemented with L-cystine) and 10% energy from fat. In the STD diet, fat was made up of 67% lard and 33% soybean oil. The ω-3 PUFA diet derived 2.3% of the total energy as ω-3 PUFA (3.3% fish oil that was comprised of 21% EPA and 49% DHA by mass; Ocean Nutrition, Dartmouth, Nova Scotia, Canada), with the balance of fat from lard and soybean oil. This fish oil dose corresponds to a human intake of approximately 5.1 g/d of DHA+EPA (calculated assuming an energy intake of 2000 kcal/d) [7,9].

### Echocardiography

LV function was assessed using a Vevo 770 High-Resolution Imaging Systems (Visual Sonics) with a 30-MHz linear array transducer (model 716). Anesthetized mice were shaved and placed supine on a warming pad. Two-dimensional cine loops and guided M-mode frames were recorded from the parasternal short and long axes. At the end of the study, all data were analyzed offline with software resident on the ultrasound system, and calculations were made to determine LV volumes as previously described. Ejection fraction was calculated as: (EDV-ESV)/EDV × 100, where EDV is the end diastolic volume and ESV is the end systolic volume. Relative wall thickness was calculated as the sum of the posterior and anterior wall thickness at end diastole divided by the end diastolic diameter. Mean wall thickness was taken as the average of the posterior and anterior wall thickness at end diastole. All calculations were made from parasternal short axis measurements.

### Metabolic and Biochemical Parameters

Free fatty acids, triglycerides, and glucose (Wako, Richmond, VA) were assessed in the serum using enzymatic spectrophotometric methods as previously described [7]. Enzyme-linked immunosorbent assays were used to measure leptin and insulin (both from Alpco Diagnostics, Salem, NH), and adiponectin (Millipore, St. Charles, MO) levels in the serum. Activities of the mitochondrial marker enzymes citrate synthase and medium chain acyl-CoA dehydrogenase (MCAD) were measured spectrophotometrically in LV myocardium [7]. Cardiac phospholipid fatty acid composition was assessed in a subset of sham animals (n = 6/group) on right ventricular homogenates by gas chromatography with a flame ionization detector according to a modification of the transesterification method as previously described [25].

For assessment of mRNA expression, frozen LV tissue was homogenized using a Bullet Blender and RNA was isolated using the RNeasy Mini Kit following the manufacturer's instructions (Qiagen, Valencia, CA). Real time RT-PCR was performed using an ABI 7900 Detection System as previously described [6]. The following genes were analyzed, using TaqMan gene expression assays (Applied Biosystems, Foster City, CA): atrial natriuretic peptide (*Nppa*, Mm01255747_g1); myosin heavy chain α (*Myh6*, Mm00440354_m1); myosin heavy chain β (*Myh7*, Mm00600555_m1); MCAD (*Acadm*, Mm00431611_m1); citrate synthase (*Cs*, Mm00466043_m1); uncoupling protein 3 (*Ucp3*, Mm01163394_m1), pyruvate dehydrogenase kinase 4 (*Pdk4*, Mm00443325_m1), and cyclophilin A (*Ppia*, Mm02342430_g1). A similar protocol was followed to isolate RNA from frozen epididymal fat samples, and RT-PCR was performed for adiponectin (*Adipoq*, Mm00456425_m1). mRNA cycle threshold values for these genes were normalized to the house-keeping gene cyclophilin A and expressed as fold increase relative to the standard chow sham group.

### Statistical Analysis

Two-way ANOVA with the Bonferonni post hoc adjustment was used to compare the effects of surgery and diet within the wild type and adiponectin-/- groups separately and to assess the interaction between surgery and dietary groups. Statistical comparisons were not made between wild type and adiponectin-/- groups. Potential differences in survival were tested using a log rank test (Sigma Stat 3.5). All data are presented as means ± SEM. P < 0.05 was accepted as statistically significant.

## RESULTS

### Myocardial Phospholipid Composition

Supplementation with ω-3 PUFA significantly altered the myocardial phospholipid fatty acid composition, with an increase in DHA and EPA content, and decreased arachidonic acid content in both WT and adiponectin-/- mice (Table 1). DHA content increased by ~70% with ω-3 PUFA supplementation, and arachidonic acid decreased by 75%. Palmitic acid was unaffected by diet, but stearic acid, oleic acid, and linoleic acid levels were all decreased in animals fed ω-3 PUFA (Table 1).

**Table 1:** Right Ventricular Phospholipid Composition in Sham Mice

	WILD TYPE		ADIPONECTIN-/-	
**Fatty Acid **(% of total)	**STD**	**ω-3 PUFA**	**STD**	**ω-3 PUFA**
Docosahexaenoic Acid (C22:6n3)	*30.5 ± 1.2*	*50.5 ± 2.2#*	*30.3 ± 1. 2*	*51.5 ± 0.9#*
Eicosapentaenoic Acid (C20:5n3)	BQL	*1.9 ± 0.2#*	BQL	*1.6 ± 0.2#*
Arachidonic Acid (C20:4n6)	*8.3 ± 0.3*	*1.8 ± 0.1#*	*9.0 ± 0.4*	*1.8 ± 0.1#*
Palmitic Acid (C16:0)	*14.6 ± 0.5*	*13.3 ± 0.6*	*13.9 ± 0.1*	*13.8 ± 0.4*
Stearic Acid (C18:0)	*17.7 ± 0.4*	*15.6 ± 0.6#*	*17.5 ± 0.2*	*15.0 ± 0.2#*
Oleic Acid (C18:1n9)	*8.8 ± 0.4*	*6.6 ± 0.6#*	*9.0 ± 0.5*	*5.6 ± 0.4#*
Linoleic Acid (C18:2n6)	*16.0 ± 0.7*	*7.7 ± 0.4#*	*16.5 ± 0.4*	*8.1 ± 0.3#*

Values are mean ± SEM; n = 6/group. # P < 0.05 vs. standard chow diet. BQL, below quantifiable limits; TAC, transverse aortic constriction; STD, standard chow.

### Left Ventricular Mass, Remodeling, and Contractile Function

**Figure 1:**
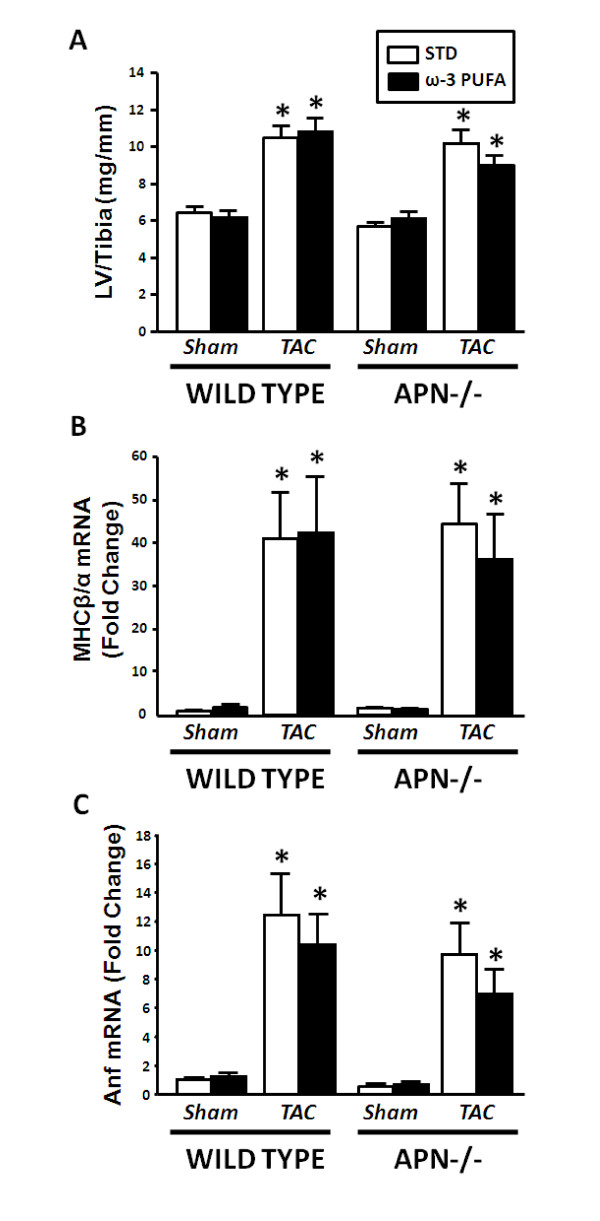
**LV mass/tibia length (A), the mRNA expression of the ratio of myosin heavy chain (MHC) β to MHCα (B), and the mRNA expression of atrial natriuretic peptide (ANF) expressed as a fraction of the sham wild type standard chow diet**. Values are mean ± SEM; n = 12-15/group. *P < 0.05 vs. sham.

There were no differences among any of the experimental groups in body mass at baseline or at the end of the study (Table 2). There was no mortality in sham animals. In TAC mice survival from the time of assignment to dietary treatment to the scheduled time of termination was unaffected by treatment with ω-3 PUFA. Survival in WT TAC mice was 71% and 72% for the STD and ω-3 PUFA diets, respectively. Similarly, treatment with ω-3 PUFA had no significant effect on survival in adiponectin-/- mice (100% and 82% for STD and ω-3 PUFA, respectively). Tail artery systolic blood pressure was significantly reduced with TAC in WT mice fed STD, but not ω-3 PUFA, and was unchanged in adiponectin-/- mice with TAC (Table 2). LV mass expressed relative to tibia length was increased to a similar extent in all TAC groups regardless of diet (Figure 1A), indicating LV hypertrophy. Atrial mass was elevated in WT mice subjected to TAC regardless of diet, and elevated in adiponectin-/- mice subjected to TAC on STD (Table 2). TAC up-regulated mRNA levels for genes previously shown to be expressed in heart failure, as seen in a 40-fold increase in the ratio for MHCβ/α in response to TAC regardless of diet in both strains of mice (Figure 1B). In addition, mRNA for ANF was significantly increased in all TAC animals compared to sham in WT and adiponectin-/- mice (Figure 1C).

**Table 2:** Body and Heart Parameters

	WILD TYPE				ADIPONECTIN-/-			
	**SHAM**		**TAC**		**SHAM**		**TAC**	
	**STD**	**ω-3 PUFA**	**STD**	**ω-3 PUFA**	**STD**	**ω-3 PUFA**	**STD**	**ω-3 PUFA**
Baseline body mass (g)	*23.2 ± 0.4*	*22.9 ± 0.3*	*22.9 ± 0.3*	*23.6 ± 0.3*	*21.7 ± 0.4*	*21.7 ± 0.5*	*21.5 ± 0.3*	*22.2 ± 0.5*
Terminal body mass (g)	*28.0 ± 0.6*	*27.6 ± 0.5*	*26.1 ± 0.1*	*25.8 ± 1.1*	*26.6 ± 0.8*	*25.5 ± 0.6*	*26.5 ± 0.7*	*26.9 ± 0.6*
Tibia length (mm)	*19.4 ± 0.2*	*19.7 ± 0.3*	*19.2 ± 0.2*	*19.6 ± 0.2*	*18.9 ± 0.3*	*18.5 ± 0.2*	*18.7 ± 0.2*	*19.1 ± 0.3*
Atrial mass/tibia length (mg·cm^-1^)	*0.73 ± 0.07*	*0.50 ± 0.04*	1.60 ± 0.29*	1.16 ± 0.13*	*0.55 ± 0.06*	*0.76 ± 0.09*	1.00 ± 0.09*	*0.93 ± 0.13*
Mean Diastolic Wall Thickness (μm)	*76.5 ± 2.8*	*74.7 ± 3.7*	*80.9 ± 4.6*	100.4 ± 3.6*#	*79.8 ± 3.8*	*84.0 ± 3.6*	114.8 ± 5.8*	109.1 ± 6.9*
Ejection Fraction (%)	*50.9 ± 2.04*	*48.8 ± 3.5*	25.9 ± 3.6*	22.9 ± 3.3*	*51.5 ± 5.1*	*61.0 ± 3.0*	*46.5 ± 3.6*	*51.8 ± 3.5*
Systolic Blood Pressure (mm Hg)	*112 ± 3*	*105 ± 3*	99 ± 2*	*100 ± 3*	*120 ± 3*	*116 ± 3*	*112 ± 3*	*117 ± 3*
Heart Rate (bpm)	*597 ± 14*	*581 ± 15*	637 ± 11*	*616 ± 10*	*604 ± 13*	*577 ± 11*	*619 ± 11*	*610 ± 14*

Values are mean ± SEM; n = 12-15/group. *P < 0.05 vs. sham; # P < 0.05 vs. standard chow diet. TAC, transverse aortic constriction; STD, standard chow. Heart rate data were obtained in conscious mice during tail cuff blood pressure measurements.

**Figure 2:**
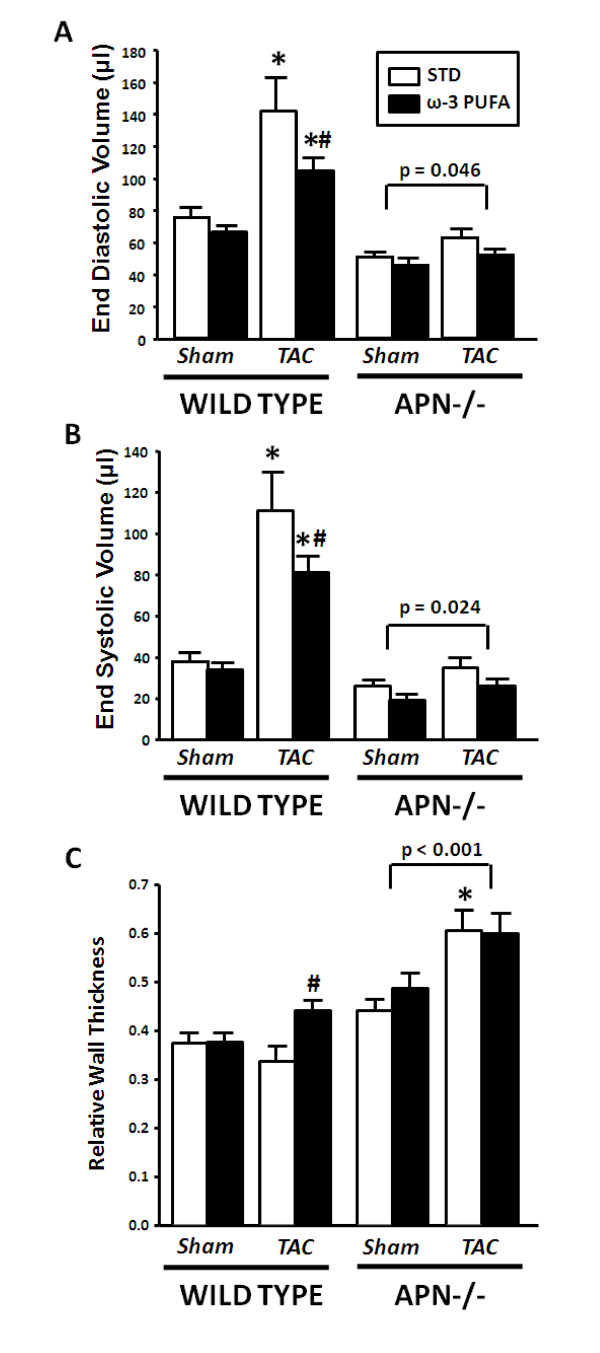
**Echocardiographic assessment of left ventricular end diastolic volume (A), end systolic volume (B), and relative wall thickness (C)**. Values are mean ± SEM; n = 12-15/group. *P < 0.05 vs. sham; # P < 0.05 vs. standard chow diet.

In WT mice TAC increased LV end diastolic volume (EDV) and end systolic volume (ESV), which was significantly blunted with ω-3 PUFA supplementation (Figure 2). In addition, the prevention in LV dilation with ω-3 PUFA treatment corresponded to an increase in wall thickness compared to TAC mice fed the STD diet (Figure 2). On the STD diet TAC resulted in a modest increase in heart rate, which was not observed in the ω-3 PUFA group (Table 2). In WT mice ejection fraction was reduced by TAC and was not affected by ω-3 PUFA supplementation (Table 2),

In adiponectin-/- mice treatment with ω-3 PUFA had no significant effects on LV volume in either sham or TAC groups, however, as we previously reported in a separate publication, these animals did not develop significant LV chamber dilation in response to TAC (Figure 2). There was a main effect of TAC on EDV and ESV (p = 0.046 and p = 0.024, respectively), but the increase was minor (Figure 2A-B). Furthermore, ejection fraction was preserved in adiponectin-/- mice subjected to TAC, with no effect of ω-3 PUFA supplementation (Table 2). Relative and mean wall thickness in adiponectin-/- mice was increased by TAC on both diets (Figure 2, Table 2).

### Mitochondrial Enzymes

**Figure 3:**
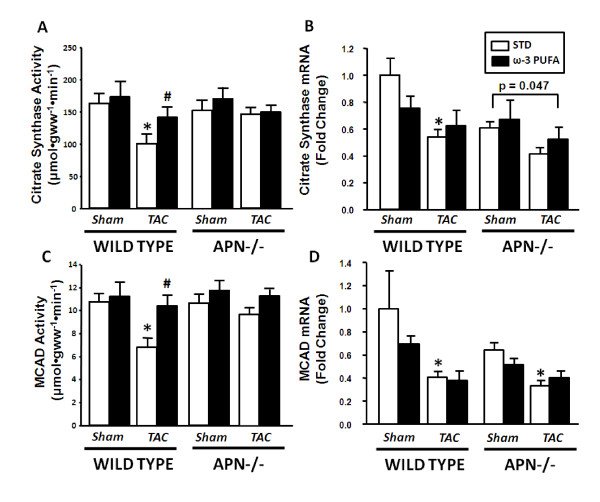
**Myocardial activity of citrate synthase (A) and medium chain acyl-CoA dehydrogenase (MCAD) (B), and the mRNA expression of MCAD (C) and citrate synthase (D)**. Values are mean ± SEM; n = 12-15/group. *P < 0.05 vs. sham; # P < 0.05 vs. standard chow diet.

In LV hypertrophy and heart failure, mitochondrial function and the fatty acid oxidation pathway are impaired [6,26]. Supplementation with ω-3 PUFA, which are ligands for PPARα [7], could activate expression of genes encoding mitochondrial enzymes, thereby reducing the impairment of myocardial energy metabolism observed with heart failure. In WT mice, there was a ~35% reduction in activities of citrate synthase and MCAD in LV myocardium with TAC, which was completely blocked by ω-3 PUFA supplementation. This effect of ω-3 PUFA was not due to activation of gene expression, as the mRNA levels of PPARα-regulated genes was not increased. PDK4 and UCP3 mRNA was significantly reduced with TAC in STD-fed mice only, and ω-3 PUFA supplementation reduced PDK4 expression in sham mice (Table 3). MCAD and citrate synthase mRNA was reduced with TAC in mice fed STD chow only (Figure 3D).

**Table 3:** Metabolic Parameters

	WILD TYPE				ADIPONECTIN-/-			
	**SHAM**		**TAC**		**SHAM**		**TAC**	
	**STD**	**ω-3 PUFA**	**STD**	**ω-3 PUFA**	**STD**	**ω-3 PUFA**	**STD**	**ω-3 PUFA**
Serum Adiponectin (μg·ml^-1^)	*7.94 ± 1.23*	*8.74 ± 1.35*	*7.72 ± 1.27*	*7.48 ± 1.23*	*1.70 ± 0.12*	*1.56 ± 0.10*	*1.50 ± 0.10*	*1.49 ± 0.10*
Serum HMW Adiponectin (μg·ml^-1^)	*0.55 ± 0.03*	*0.78 ± 0.08#*	*0.63 ± 0.05*	*0.73 ± 0.08*	*0.18 ± 0.03*	*0.18 ± 0.03*	*0.19 ± 0.02*	*0.19 ± 0.03*
Adiponectin mRNA in Epididymal Fat	*1.00 ± 0.17*	*1.10 ± 0.10*	*1.04 ± 0.14*	*1.18 ± 0.27*	*0*	*0*	*0*	*0*
PDK4 mRNA in LV	*1.00 ± 0.35*	*0.30 ± 0.03#*	0.29 ± 0.04*	*0.29 ± 0.04*	*0.27 ± 0.03*	*0.40 ± 0.11*	*0.37 ± 0.08*	*0.48 ± 0.14*
UCP3 mRNA in LV	*1.00 ± 0.14*	*0.88 ± 0.13*	0.39 ± 0.07*	*0.47 ± 0.12*	*0.89 ± 0.12*	*0.96 ± 0.11*	*0.49 ± 0.09*	*0.59 ± 0.10*
Serum Glucose (mM)	*12.7 ± 0.6*	*11.6 ± 0.8*	*12.6 ± 1.0*	*11.4 ± 0.9*	*13.0 ± 0.9*	*12.7 ± 0.7*	*11.7 ± 0.4*	*13.1 ± 1.1*
Serum FFA (mM)	*0.69 ± 0.05*	*0.55 ± 0.04*	*0.76 ± 0.14*	*0.55 ± 0.07*	*0.82 ± 0.08*	*0.47 ± 0.06*	*0.61 ± 0.07*	*0.52 ± 0.08*
Serum Insulin (ng·ml^-1^)	*0.66 ± 0.16*	*0.90 ± 0.20*	*0.66 ± 0.16*	*0.48 ± 0.10*	*0.84 ± 0.23*	*0.54 ± 0.11*	*0.60 ± 0.10*	*0.34 ± 0.10*
Serum Leptin (ng·ml^-1^)	*2.56 ± 0.51*	*2.80 ± 0.70*	*1.62 ± 0.37*	1.18 ± 0.28*	*3.83 ± 1.50*	*2.28 ± 0.82*	*1.85 ± 0.43*	*0.78 ± 0.14*
Serum Triglycerides (mg·ml^-1^)	*0.26 ± 0.05*	*0.25 ± 0.06*	*0.25 ± 0.07*	*0.28 ± 0.06*	*0.40 ± 0.09*	*0.23 ± 0.08*	*0.30 ± 0.04*	*0.25 ± 0.06*

Values are mean ± SEM; n = 12-15/group. *P < 0.05 vs. sham, #P < 0.05 vs. standard chow diet. HMW, high molecular weight; PDK4, pyruvate dehydrogenase kinase 4; UCP3, uncoupling protein 3; FFA, free fatty acids; LV, left ventricle; TAC, transverse aortic constriction; STD, standard chow. mRNA data are expressed as fold change relative to the mean of the wild type sham standard chow group.

In adiponectin-/- mice the activities of MCAD and citrate synthase were unaffected by surgery or diet (Figure 3A-B). There were no differences in PDK4 or UCP3 expression within adiponectin-/- mice, but MCAD mRNA was reduced by TAC with STD chow only (Figure 3D). Citrate synthase was decreased in adiponectin-/- mice as a main effect of surgery (p = 0.047) (Figure 3C).

### Metabolic Parameters

In WT mice, total adiponectin was not affected by diet or surgery, but ω-3 PUFA significantly increased high molecular weight adiponectin in sham animals and as a main effect (p = 0.011). However, this effect of ω-3 PUFA was modest in the TAC group (~16%). Total serum adiponectin and high molecular weight adiponectin did not change with diet or surgery in adiponectin-/- mice (Table 3). The mRNA level of adiponectin was undetectable in the epididymal fat pads of all adiponectin-/- mice and unaltered by diet or surgery in WT mice (Table 3).

Serum glucose, free fatty acids, triglycerides, and insulin were not different among any of the groups in either strain (Table 3). Serum leptin was not different among adiponectin-/- groups, but was significantly reduced in the WT TAC compared to sham ω-3 PUFA-fed mice.

## Discussion

The main novel finding of the present study is that treatment with ω-3 PUFA can prevent down-regulation in the activity of mitochondrial enzymes under conditions of severe pressure overload despite no diminution in LV hypertrophy or the classic up-regulation of the mRNA for fetal genes. Importantly, the maintenance in mitochondrial enzyme activity corresponded with attenuation of LV chamber expansion. Lastly, ω-3 PUFA supplementation did not increase adiponectin in TAC animals, suggesting that the protective effects of ω-3 PUFA occur independent of up-regulation of circulating adiponectin.

In the present study we observed a protective effect of ω-3 PUFA in WT mice, with an attenuation of the increase in end diastolic and systolic volumes with TAC and maintenance of cardiac mitochondrial enzyme function. In our previous studies in rats with a similar dose of ω-3 PUFA we observed a large increase (~70%) in total circulating adiponectin [6,7,9]. In the current study we did not see the expected increase in total serum adiponectin in WT mice with ω-3 PUFA supplementation. However, we did observe a small non-significant 16% increase in high molecular weight adiponectin in the serum. The lack of effect of ω-3 PUFA on total adiponectin could be due to regulation of adiponectin formation and secretion, thereby changing the amount of the various molecular weight isoforms. In addition, there could be differences between species. Most reports with fish oil supplementation indicate an up-regulation of the expression and secretion of adiponectin in rats [6-8] and humans [27]; however, there are reports where adiponectin is unaffected by fish oil supplementation in rats and mice [28,29]. In a study in mice fed a diet substituted with various DHA analogs, serum adiponectin was either reduced or unchanged [29]. Clearly, additional research is needed to address the specific role of ω-3 PUFA in adiponectin regulation.

Long chain fatty acids, including ω-3 PUFA are endogenous ligands for PPARα [30], which may prevent the decline in PPARα activation and mitochondrial function that is commonly observed in advanced heart failure [31]. In the present investigation we show preservation of mitochondrial enzyme activities with dietary ω-3 PUFA supplementation after TAC. However, the mRNA for genes encoding the mitochondrial proteins citrate synthase, MCAD, PDK4 and UCP3 does not closely mirror the changes observed in activities of citrate synthase and MCAD (Table 3 and Figure 3). The mRNA for all four genes decreased with TAC in WT mice fed STD, but was not increased by ω-3 PUFA feeding. This discrepancy between gene expression and enzyme activity has been previously reported in other models of heart failure and pathological cardiac hypertrophy [10,32]. Thus, it does not appear that ω-3 PUFA preferentially activate PPARα under physiological conditions to confer cardioprotection in the setting of heart failure. ω-3 PUFA supplementation may be maintaining mitochondrial enzyme activity via changes in mitochondrial membrane composition, as we observed an increase in DHA and decrease in arachidonic acid, which may favorably alter mitochondrial function and structure, as previously suggested [33].

The effects of ω-3 PUFA on structural and metabolic LV remodeling were markedly different compared to STD-fed controls in each strain. The inherent differences in the response of the LV to pressure overload between the two strains of mice have been detailed in our previous report [22]. We observed the expected chamber dilation and reduction in ejection fraction after TAC in WT mice, but severe concentric hypertrophy with maintenance of ejection fraction in adiponectin-/- mice. In the present investigation we found that WT mice experienced LV dilation that was attenuated by ω-3 PUFA supplementation, but there was no effect of ω-3 PUFA on LV remodeling in adiponectin-/- mice. In addition, the WT mice fed STD displayed a decrease in the activity of mitochondrial oxidative enzymes that classically occurs in advanced LV hypertrophy and heart failure [34], but the activities were maintained with ω-3 PUFA supplementation. In the adiponectin-/- mice, there was no effect of TAC or dietary ω-3 PUFA on mitochondrial enzyme activities. Taken together, it appears that the beneficial effects of ω-3 PUFA on the heart are not dependent on an increase in adiponectin.

The effects of ω-3 PUFA supplementation on phospholipid composition in cardiac tissue have been well-documented in humans [35], rats [7,9], dogs [36], and mice [37]. Notably, mice have higher DHA levels in cardiac phospholipids without dietary ω-3 PUFA (~30% of total phospholipid fatty acid; Table 1) [37] compared to humans and dogs (~1%) [35,36] or rats (~8%) [7,9]. In the present study ω-3 PUFA supplementation increased the DHA content of membranes in mice to approximately 50% of total fatty acids, while a low level of supplementation increases DHA content to approximately 2-3% in humans [35]. The pronounced difference in membrane DHA content between humans and mice limits the application of these results from mice to human disease.

In summary, treatment with ω-3 PUFA prevented LV chamber expansion and down-regulation of the activity of mitochondrial enzymes under conditions of severe pressure overload despite no diminution in LV hypertrophy or mRNA markers of heart failure. ω-3 PUFA supplementation maintained cardiac mitochondrial enzyme activity, which corresponded with prevention of LV chamber expansion. These effects occurred despite no ω-3 PUFA-induced increase in adiponectin, suggesting that the protective effects of ω-3 PUFA occur independent of up-regulation of circulating adiponectin.

## Conflict of interest

The authors declare that they have no competing interests.

## Authors' contributions

KMO designed the study, performed the physiological and biochemical measurements, analyzed the data and wrote the manuscript. DJC assisted in the design of the study and performed all animal surgeries. RJK performed biochemical analyses. PAH performed biochemical analyses. BL performed extensive preliminary studies and assisted with the study design. K W assisted in the design of the study, data analysis, and writing the manuscript. CDR performed the phospholipid analysis, and assisted in data analysis and writing the manuscript. WCS conceived and designed the study, analyzed the data and wrote the manuscript. All authors read and approved the final manuscript
